# Erratum: Paolo Lauriola et al., Advancing Global Health through Environmental and Public Health Tracking, *Int. J. Environ. Res. Public Health* 2020, *17*, 1976

**DOI:** 10.3390/ijerph17228568

**Published:** 2020-11-18

**Authors:** Paolo Lauriola, Helen Crabbe, Behrooz Behbod, Fuyuen Yip, Sylvia Medina, Jan C. Semenza, Sotiris Vardoulakis, Dan Kass, Ariana Zeka, Irma Khonelidze, Matthew Ashworth, Kees de Hoogh, Xiaoming Shi, Brigit Staatsen, Lisbeth E. Knudsen, Tony Fletcher, Danny Houthuijs, Giovanni S. Leonardi

**Affiliations:** 1 National Research Council, Institute of Clinical Physiology, Unit of Environmental Epidemiology and Disease Registries, 56124 Pisa, Italy; 2 Centre for Radiation, Chemical and Environmental Hazards, Public Health England, Didcot, Oxon OX11 0RQ, UK; helen.crabbe@phe.gov.uk (H.C.); Tony.fletcher@phe.gov.uk (T.F.); Giovanni.leonardi@phe.gov.uk (G.S.L.); 3 Centre for Medical Education, Cardiff University, Cardiff CF14 4XW, UK; behbodb@cardiff.ac.uk; 4 Centers for Disease Control and Prevention, Atlanta, GA 30341, USA; fay1@cdc.gov; 5 Direction of Environmental and Occupational Health, Santé Publique France, 94415 Saint Maurice, France; Sylvia.MEDINA@santepubliquefrance.fr; 6 Scientific Assessment Section, European Centre for Disease Prevention and Control, 169 73 Solna, Sweden; JanC.Semenza@ecdc.europa.eu; 7 National Centre for Epidemiology and Population Health, Research School of Population Health, Australian National University, Canberra 2601, Australia; sotiris.vardoulakis@anu.edu.au; 8 Vital Strategies, New York, NY 10005, USA; dkass@vitalstrategies.org; 9 Environmental Health and Epidemiology, Brunel University, London UB8 3PH, UK; Ariana.Zeka@brunel.ac.uk; 10 Medical University Centre “Nene Teresa”, Rruga e Dibres, #370 Tirana, Albania; 11 National Center for Disease Control and Public Health, 0198 Tbilis, Georgia; i.khonelidze@ncdc.ge; 12 Institute of Environmental Science and Research Limited, Kenepuru, Porirua 5240c, New Zealand; Matthew.Ashworth@esr.cri.nz; 13 Swiss Tropical and Public Health Institute, 4051 Basel, Switzerland; c.dehoogh@swisstph.ch; 14 University of Basel, 4001 Basel, Switzerland; 15 National Institute of Environmental Health, Chinese Center for Disease Control and Prevention, Beijing 100021, China; shixm@chinacdc.cn; 16 National Institute for Public Health and the Environment, 3720BA Bilthoven, The Netherlands; Brigit.staatsen@rivm.nl (B.S.); Danny.Houthuijs@rivm.nl (D.H.); 17 Department of Public Health, Denmark University of Copenhagen, 1353 Copenhagen, Denmark; liek@sund.ku.dk; 18 London School of Hygiene and Tropical Medicine, London WC1E 7HT, UK

Due to an error during production, some contents of Table 1 are missing in the published paper [1].

**Incorrect:**

**Table 1:** Tracking activities around the globe that are collaborating in an international EPHT network (INPHET).

**Country.**	**Name/Subjects**	**Leading Institution**
**EPHT SYSTEMS**		
**Australia** [48–51]	The Chinese Environmental Public Health Tracking (CEPHT) project started in September 2015 operated by the National Institute of Environmental Health, Chinese Center for Disease Control and Prevention (NIEH, China CDC), developing CEPHT’s electronic data tracking system. Twenty-nine local Chinese CDCs participate by reporting environmental hazard and health effects data through the electronic system.	National Institute of Environmental Health (NIEH, China CDC)
**EPHT-EQUIVALENT SYSTEMS**		
***North America***		
**Canada**	Environmental Public Health Program: Focus on outdoor air quality, water quality and soil contaminants. Health databases include emergency department visits, hospital admissions, cancer and mortality data [52] Acute Care Enhanced Surveillance (ACES) is a real-time syndromic surveillance system with temporal and spatial capabilities that enables public health to be better informed on the health of the community. ACES’ syndromic surveillance capabilities are useful in a variety of situations, including public health emergencies, such as extreme weather events [53]	Canadian Urban Environmental Health Research Consortium (CANUE) The ACES system is maintained by KFL&A Public Health and is funded by the Ministry of Health and Long Term Care
***Europe***		
**European Union**	European Environment and Epidemiology (E3) Network [54], providing access to climatic/environmental geospatial data for epidemiologic analysis currently collected and analysed by a variety of European agencies, public health institutes, and research organisations The system is an interactive database composed of country-level indicators and regional assessments [55]. ENHIS indicators provide information on exposures, health outcomes and policy actions related to the environment and health priority areas for the European Region known as Regional Priority Goals (RPGs) The initiative is coordinating and advancing human biomonitoring in Europe. HBM4EU is generating evidence of the actual exposure of citizens to chemicals and the possible health effects to support policymaking [56].	European Centre for Disease Prevention and Control (ECDC) *Environment and Health Information System* (ENHIS) *European Human Biomonitoring Initiative* (HBM4EU)
**Italy**	The main activity that has characteristics of ongoing surveillance is on “Mesothelioma and Asbestos exposure tracking” [57]. There are also several studies with the potential to become ongoing surveillance programmes. Sentieri [58], which describes the health profile of populations living in contaminated sites, and provides elements for the design of ongoing monitoring, that could have value across several countries [59,60] Moniter [61], dealing with incinerator risks EpiAir [62] for air pollution surveillance Environmental Health Task Force (EHTF) appointed by the Ministry of Health (MoH), is underpinning a strategy to develop a framework which includes local and national resources (underway).	National Institute of Health National Institute of Health ARPA-ER DEP-ARPAP EHTTF-MoH
**The Netherlands**	Small Area Health Studies using health registries [63] Statistics Netherlands (CBS, Ministry Economic Affaires), Strategic Project Health Care [63] Lung cancer in the IJmond region in relation to cadmium exposure [64] Aircraft noise annoyance in the vicinity of Amsterdam Schiphol Airport [65]	National Institute of Public Health and the Environment (RIVM)
***Eurasia***		
**Georgia** [63]	A Multiple Indicator Cluster Surveys (MICS) on a representative sample of children to study several demographic and health aspects over the last ten years. Starting from lead biomonitoring in children, a national surveillance programme is underway. Considering the geographical and geopolitical situation of the region, the experience in Georgia may lead to an initiative in collaboration with neighbouring countries, with the potential to establish a regional Eurasia hub for EPHT, based on a network established at a recent EU funded TAIEX workshop.	National Center for Disease Control and Public Health (NCDC-PH)
**Pacific Region**		
**New Zealand**	A system reports on a range of environmental hazards, with the additional synthesis of the environmental burden of disease for second-hand smoke exposure. Also working on developing environmental burden of disease reports for ultraviolet light exposure and lead (Pb) exposure [66,67] Reporting provides weekly communicable disease surveillance and outbreak surveillance reporting to New Zealand regional health authorities.	Massey University Institute of Environmental Science and Research [68]
**Kingdom of Tonga** [69]	Vector and vector-borne disease surveillance that includes meteorological and Southern Ocean oscillation index data, and relates to the climate change monitoring data collected in Tonga Water quality, water-borne disease and infection monitoring	Ministry of Health, Kingdom of Tonga
**South Pacific Region** [70]	The Pacific Public Health Surveillance Network (PPHSN) is a voluntary network of countries and organisations dedicated to the promotion of public health surveillance and appropriate response to the health challenges of 22 Pacific Island countries and territories (PICTs). It includes six service networks: PacNet, LabNet, EpiNet, PICNet, the Pacific Syndromic Surveillance System and the Strengthening Health Interventions in the Pacific-Data for Decision Making (SHIP-DDM) capacity development programme.	Secretariat for the Pacific Community (SPC)

**Correct:**

**Table 1:** Tracking activities around the globe that are collaborating in an international EPHT network (INPHET).

**Country**	**Name/Subjects**	**Leading Institution**
**EPHT SYSTEMS**		
**Australia** [48]	Currently developing a strategy for an Environmental Health Tracking System in Victoria. A Driving Force-Pressure-Environmental Condition-Health Impact-Action (DPEHA) conceptual framework is proposed for the proposed Victorian EHTS.	Environmental Protection Agency (EPA) Victoria’s Environmental Public Health Unit
**China** [49–51]	The Chinese Environmental Public Health Tracking (CEPHT) project started in September 2015 operated by the National Institute of Environmental Health, Chinese Center for Disease Control and Prevention (NIEH, China CDC), developing CEPHT’s electronic data tracking system. Twenty-nine local Chinese CDCs participate by reporting environmental hazard and health effects data through the electronic system.	National Institute of Environmental Health (NIEH, China CDC)
**EPHT-EQUIVALENT SYSTEMS**		
***North America***		
**Canada**	Environmental Public Health Program: Focus on outdoor air quality, water quality and soil contaminants. Health databases include emergency department visits, hospital admissions, cancer and mortality data [52] Acute Care Enhanced Surveillance (ACES) is a real-time syndromic surveillance system with temporal and spatial capabilities that enables public health to be better informed on the health of the community. ACES’ syndromic surveillance capabilities are useful in a variety of situations, including public health emergencies, such as extreme weather events [53].	Canadian Urban Environmental Health Research Consortium (CANUE) The ACES system is maintained by KFL&A Public Health and is funded by the Ministry of Health and Long Term Care
***Europe***		
**European Union**	European Environment and Epidemiology (E3) Network [54], providing access to climatic/environmental geospatial data for epidemiologic analysis currently collected and analysed by a variety of European agencies, public health institutes, and research organisations The system is an interactive database composed of country-level indicators and regional assessments [55]. ENHIS indicators provide information on exposures, health outcomes and policy actions related to the environment and health priority areas for the European Region known as Regional Priority Goals (RPGs) The initiative is coordinating and advancing human biomonitoring in Europe. HBM4EU is generating evidence of the actual exposure of citizens to chemicals and the possible health effects to support policymaking [56].	European Centre for Disease Prevention and Control (ECDC) *Environment and Health Information System* (ENHIS) *European Human Biomonitoring Initiative* (HBM4EU)
**Italy**	The main activity that has characteristics of ongoing surveillance is on “Mesothelioma and Asbestos exposure tracking” [57]. There are also several studies with the potential to become ongoing surveillance programmes Sentieri [58], which describes the health profile of populations living in contaminated sites, and provides elements for the design of ongoing monitoring, that could have value across several countries [59,60] Moniter [61], dealing with incinerator risks EpiAir [62] for air pollution surveillance Environmental Health Task Force (EHTF) appointed by the Ministry of Health (MoH), is underpinning a strategy to develop a framework which includes local and national resources (underway).	National Institute of Health National Institute of Health ARPA-ER DEP-ARPAP EHTTF-MoH
**The Netherlands**	Small Area Health Studies using health registries [63] Statistics Netherlands (CBS, Ministry Economic Affaires), Strategic Project Health Care [63] Lung cancer in the IJmond region in relation to cadmium exposure [64] Aircraft noise annoyance in the vicinity of Amsterdam Schiphol Airport [65].	National Institute of Public Health and the Environment (RIVM)
***Eurasia***		
**Georgia** [63]	A Multiple Indicator Cluster Surveys (MICS) on a representative sample of children to study several demographic and health aspects over the last ten years. Starting from lead biomonitoring in children, a national surveillance programme is underway Considering the geographical and geopolitical situation of the region, the experience in Georgia may lead to an initiative in collaboration with neighbouring countries, with the potential to establish a regional Eurasia hub for EPHT, based on a network established at a recent EU funded TAIEX workshop.	National Center for Disease Control and Public Health (NCDC-PH)
**Pacific Region**		
**New Zealand**	A system reports on a range of environmental hazards, with the additional synthesis of the environmental burden of disease for second-hand smoke exposure. Also working on developing environmental burden of disease reports for ultraviolet light exposure and lead (Pb) exposure [66,67] Reporting provides weekly communicable disease surveillance and outbreak surveillance reporting to New Zealand regional health authorities.	Massey University Institute of Environmental Science and Research [68]
**Kingdom of Tonga** [69]	Vector and vector-borne disease surveillance that includes meteorological and Southern Ocean oscillation index data, and relates to the climate change monitoring data collected in Tonga Water quality, water-borne disease and infection monitoring.	Ministry of Health, Kingdom of Tonga
**South Pacific Region** [70]	The Pacific Public Health Surveillance Network (PPHSN) is a voluntary network of countries and organisations dedicated to the promotion of public health surveillance and appropriate response to the health challenges of 22 Pacific Island countries and territories (PICTs). It includes six service networks: PacNet, LabNet, EpiNet, PICNet, the Pacific Syndromic Surveillance System and the Strengthening Health Interventions in the Pacific-Data for Decision Making (SHIP-DDM) capacity development programme.	Secretariat for the Pacific Community (SPC)

## References

[1] Lauriola P., Crabbe H., Behbod B., Yip F., Medina S., Semenza J.C., Vardoulakis S., Kass D., Zeka A., Khonelidze I. (2020). Advancing Global Health through Environmental and Public Health Tracking. Int. J. Environ. Res. Public Health.

